# Clinical and pathophysiological perspectives on haemostasis, inflammation, and thrombo-haemorrhagic risk in cirrhosis

**DOI:** 10.17179/excli2026-9622

**Published:** 2026-08-17

**Authors:** Alberto Zanetto, Kymentie Ferdinande, Marco Senzolo

**Affiliations:** 1 Department of Surgery, Oncology and Gastroenterology, University of Padova, Padova, Italy; 2 Gastroenterology, Unit of Vascular Liver Diseases and Treatment of Portal Hypertension, Padova University Hospital, European Reference Network on Rare Liver Disorders (ERN Rare-Liver), Padova, Italy

**Keywords:** haemostasis, inflammation, immunohaemostasis, immunothrombosis, disease progression, cirrhosis, procedure-related bleeding, portal vein thrombosis, venous thromboembolism

## Abstract

Haemostasis in cirrhosis is a dynamic process characterised by a fragile equilibrium. Haemostatic alterations become more pronounced during acute decompensation and acute-on-chronic liver failure, conditions driven by increasing systemic inflammation. When compounded by bacterial infection or acute kidney injury, additional alterations may emerge, predisposing to bleeding and thrombosis. Recent data indicate an interplay between haemostasis and inflammation in chronic liver disease, with implications for disease progression and thrombo-haemorrhagic complications. In this review, we discuss evolving knowledge across four interconnected domains. First, we summarise novel insights into the haemostatic status in cirrhosis derived from functional assays assessing platelet aggregation, total thrombus formation, thrombin and plasmin generation. Second, we examine the interplay between haemostasis and inflammation, focusing on immunohaemostasis, immunothrombosis, and coagulation-driven fibrogenesis as contributors to disease progression, with altered gut-liver axis signalling as an important upstream trigger. Third, we review evolving concepts in portal vein thrombosis and venous thromboembolism, highlighting accumulating evidence that portal vein thrombosis may represent a vascular remodelling disorder characterised by endothelial-to-mesenchymal transition, driven by portal hypertension-induced low shear stress, inflammation, infection, and gut-derived inflammatory stimuli, establishing a microenvironment permissive to secondary thrombus formation. Finally, we discuss procedure-related bleeding risk assessment, underscoring the need for a multidimensional framework integrating procedure- and patient-related factors, clinical trajectory, systemic inflammation, and functional haemostatic testing. These advances reinforce an integrated paradigm in which haemostasis, inflammation, and liver disease progression are highly interconnected. Improved understanding of this interface may enhance risk stratification and identify novel therapeutic targets in advanced chronic liver disease.

See also the graphical abstract(Fig. 1).

## Abbreviations

**AASLD**, American Association for the Study of Liver Diseases; **ACLF**, acute-on-chronic liver failure; **AD**, acute decompensation; **ADAMTS13**, a disintegrin and metalloproteinase with thrombospondin type 1 motif member 13; **ADP**, adenosine diphosphate; **AKI**, acute kidney injury; **AUC**, area under the curve; **AUROC**, area under the receiver operating characteristic curve; **CiThroModel**, Cirrhosis Thrombosis Model; **CLT**, clot lysis time; **CRP**, C-reactive protein; **CXCL1**, chemokine (C-X-C motif) ligand 1; **DLL4**, Delta-like ligand 4; **DOACs**, direct oral anticoagulants; **EASL**, European Association for the Study of the Liver; **EndMT**, endothelial-to-mesenchymal transition; **ETP**, endogenous thrombin potential; **FVIII**, factor VIII; **GPIIb/IIIa**, glycoprotein IIb/IIIa; **HCC**, hepatocellular carcinoma; **HMW**, high-molecular-weight; **HR**, hazard ratio; **HSCs**, hepatic stellate cells; **HVPG**, hepatic venous pressure gradient; **ICAM-1**, intercellular adhesion molecule-1; **IL-6**, interleukin-6; **INR**, international normalised ratio; **LMW**, low-molecular-weight; **LMWH**, low-molecular-weight heparin; **LPS**, lipopolysaccharide; **LSECs**, liver sinusoidal endothelial cells; **LTA**, light transmission aggregometry; **MASLD**, metabolic dysfunction-associated steatotic liver disease; **MASH**, metabolic dysfunction-associated steatohepatitis; **MCF**, maximum clot firmness; **miR-21-5p**, microRNA-21-5p; **miR-146a-5p**, microRNA-146a-5p; **miR-155-5p**, microRNA-155-5p; **miRNA**, microRNA; **NETs**, neutrophil extracellular traps; **NLRP3**, NOD-like receptor family pyrin domain containing 3; **NO**, nitric oxide; **OR**, odds ratio; **PAI-1**, plasminogen activator inhibitor-1; **PAR**, protease-activated receptor; **PAR-1**, protease-activated receptor 1; **PAR-2**, protease-activated receptor 2; **PAR-4**, protease-activated receptor 4; **PC**, protein C; **PG**, plasmin generation; **PLT ratio**, platelet aggregation-to-platelet count ratio; **PPP**, platelet-poor plasma; **PPP-TG**, platelet-poor plasma thrombin generation; **PRP**, platelet-rich plasma; **PN-1**, platelet protease nexin-1; **PVECs**, portal vein endothelial cells; **PVT**, portal vein thrombosis; **RePLTs**, reticulated platelets; **ROTEM**, rotational thromboelastometry; **TAFI**, thrombin-activatable fibrinolysis inhibitor; b**TEG**, thromboelastography; **TG**, thrombin generation; **TG/PG ratio**, thrombin-to-plasmin generation ratio; **TIPS**, transjugular intrahepatic portosystemic shunt; **TLR4**, Toll-like receptor 4; **TNF-α**, tumour necrosis factor alpha; **tPA**, tissue-type plasminogen activator; **TPO-RAs**, thrombopoietin receptor agonists; **uPA**, urokinase-type plasminogen activator; **ULVWM**, ultra-large von Willebrand multimers; **VEGF**, vascular endothelial growth factor; **VKAs**, vitamin K antagonists; **VTE**, venous thromboembolism; **VWF**, von Willebrand factor; **VWF:Ag**, von Willebrand factor antigen; **WB-TG**, whole-blood thrombin generation


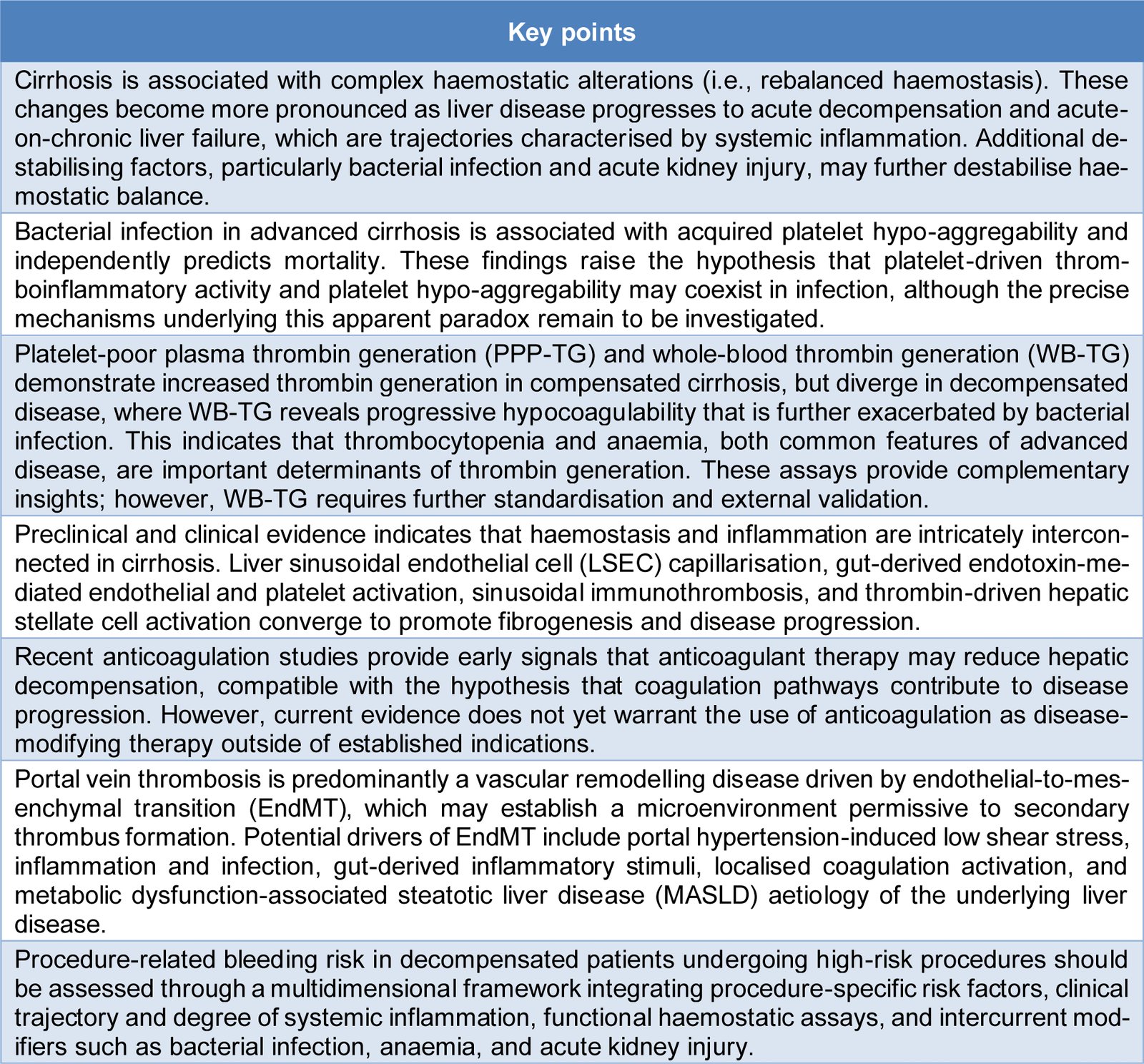


## Introduction

Haemostasis in cirrhosis is a highly dynamic and complex process. An emerging topic in haemostasis in cirrhosis is the bidirectional interplay between haemostasis and inflammation, with potential implications for disease progression and thrombo-haemorrhagic complications. In this paper, we review recent advances in four key domains: (i) haemostatic rebalance in cirrhosis, (ii) the interplay between haemostasis, inflammation, and disease progression, (iii) portal vein thrombosis and venous thromboembolism, and (iv) procedure-related bleeding; focusing on novel insights in the field. We also outline key knowledge gaps and research questions to be studied in the coming years.

## Haemostatic Rebalance in Cirrhosis

### Platelets

Primary haemostasis in cirrhosis is characterised by the interplay between thrombocytopenia, alterations in platelet function, and changes in the platelets' adhesion, driven by increased von Willebrand factor (VWF) and its principal regulator ADAMTS13 (Zanetto et al., 2024[109]). Thrombocytopenia and platelet dysfunction have historically been considered as primary determinants of bleeding tendency in decompensated cirrhosis (Northup et al., 2021[68]; Zermatten et al., 2020[121]). This concept largely originated from studies using platelet-rich plasma (PRP) and light transmission aggregometry (LTA), which suggested markedly impaired platelet aggregation. However, subsequent clinical investigations have challenged the presumed association between thrombocytopenia and bleeding risk, showing no increase in procedural bleeding, even in decompensated cirrhosis and with a platelet count below 50 × 10^9^/L (Roberts et al., 2022[82]; Zanetto et al., 2024[109]). Functional platelet testing is inherently limited by the confounding effects of thrombocytopenia, and PRP-based measurements do not accurately reflect *in vivo* haemostasis (Zanetto et al., 2024[109]). Earlier work using PRP-based LTA, in which control samples were diluted with Tyrode buffer to match thrombocytopenic platelet counts of patients with cirrhosis, demonstrated increased platelet aggregation in patients with cirrhosis compared with count-matched controls and identified endotoxin-mediated platelet activation via Toll-like receptor 4, with increased thromboxane B2 production and P-selectin expression, as a potential mechanism (Caldwell and Lisman, 2017[11]; Raparelli et al., 2017[77]). However, this approach has methodological limitations, as dilution of healthy control samples alters the plasma milieu and therefore may not accurately reflect *in vivo* conditions (Germanovich et al., 2018[34]; Ichikawa et al., 2022[44]; Raparelli et al., 2017[77]; Schols et al., 2008[84]; Watts et al., 1983[100]). To address these limitations, whole-blood ADP-induced platelet aggregation normalised to platelet count (the PLT ratio) was evaluated using impedance aggregometry (Multiplate®). The PLT ratio provides a continuous measure of platelet aggregation normalised for platelet count, allowing assessment of per-platelet aggregability independent of thrombocytopenia while preserving the physiological milieu, including VWF, red blood cells, and plasma proteins (Sharma et al., 2026[86]; Zanetto et al., 2022[104] ). The PLT ratio was significantly increased in cirrhosis compared with chronic hepatitis and healthy controls, increased in parallel with disease severity, and was highest among patients with decompensated cirrhosis independently from the platelet count (Zanetto et al., 2022[104]). On multivariate analysis, the PLT ratio was independently associated with further decompensation, transplantation, and death (Zanetto et al., 2022[104]). These findings extend earlier observations that platelet aggregation per platelet is increased, rather than decreased, in decompensated cirrhosis.

Elevated plasma levels of VWF have been proposed to counterbalance thrombocytopenia in cirrhosis (Tripodi and Mannucci, 2011[91]). VWF antigen (VWF:Ag) is markedly increased and rises progressively with disease severity (Ferlitsch et al., 2012[32]; Lisman et al., 2022[52]). In stable cirrhosis, a shift in VWF multimer distribution is observed, characterised by a relative decrease in high-molecular-weight (HMW) multimers and a corresponding increase in low-molecular-weight (LMW) multimers (Abdoul et al., 2026[1]; Palyu et al., 2018[69]). HMW VWF multimers represent the most biologically active forms of VWF and are particularly important for platelet adhesion and aggregation under conditions of high shear stress. ADAMTS13 activity is relatively preserved in absolute terms in stable cirrhosis, yet is insufficient relative to the elevated VWF substrate load. The depletion of HMW VWF multimers despite relatively preserved ADAMTS13 activity suggests contributions from non-ADAMTS13 proteolytic pathways, as well as consumptive loss through intrahepatic microthrombus formation (Groeneveld et al., 2021[37]; Palyu et al., 2018[69]; Simbrunner et al., 2023[87]). With disease progression, ADAMTS13 activity declines significantly, a process accentuated by systemic inflammation and bacterial translocation (Reuken et al., 2015[79]; Takaya et al., 2023[90]). In a subset of patients with acute decompensation and severely reduced ADAMTS13 activity, ultra-large VWF multimers (ULVWM) accumulate in the circulation, and platelet adhesion is markedly enhanced in the presence of plasma from these patients (Palyu et al., 2018[69]). Recent work has identified plasmin-mediated VWF cleavage as an alternative proteolytic pathway that is significantly upregulated in decompensated cirrhosis and acute-on-chronic liver failure (ACLF), but not in stable disease (El Otmani et al., 2025[26]). Plasmin-cleaved VWF correlated with D-dimer levels, suggesting a link between enhanced fibrinolytic activation and increased fibrin turnover in advanced disease, which may reflect ongoing microvascular thrombosis (El Otmani et al., 2025[26]). However, this alternative proteolytic pathway does not appear to fully compensate for the loss of ADAMTS13 function, as evidenced by the presence of ULVWM and enhanced platelet adhesion in a subset of patients with acute decompensation (Palyu et al., 2018[69]). The platelet-VWF axis in cirrhosis is governed by stage-dependent and incompletely characterised mechanisms, and its net contribution to haemostatic balance requires further investigation.

The aforementioned assays assess components of primary haemostasis under static conditions and consequently do not capture shear-dependent VWF-GPIb-mediated platelet adhesion and thrombus growth, processes that are central to primary haemostasis *in vivo* and particularly relevant in cirrhosis given the profound alterations in the platelet-VWF-ADAMTS13 axis (Zermatten et al., 2020[121]). Microfluidic flow-based techniques have emerged as complementary tools for investigating platelet mechanobiology under physiological shear, enabling real-time assessment of platelet adhesion, activation, and thrombus propagation on biologically relevant surfaces (Abdoul et al., 2026[1]; Mansouritorghabeh et al., 2025[58]). Among these, the Total Thrombus-formation Analysis System (T-TAS) is a commercially available microchip-based flow chamber device that quantifies whole-blood thrombus formation under defined shear rates, integrating the contributions of platelet count, platelet function, and VWF-mediated adhesion into a single readout (Mansouritorghabeh et al., 2025[58]). In a recent small study, T-TAS demonstrated markedly impaired thrombus formation in cirrhosis, with significantly prolonged occlusion times and reduced area under the curve, worsening with increasing Child-Pugh score, and were not compensated by elevated VWF levels (Abdoul et al., 2026[1]). However, as a shear-dependent whole-blood assay, T-TAS output is inherently influenced by platelet count and haematocrit and therefore reflects ove1rall primary haemostatic capacity under flow rather than intrinsic per-platelet reactivity (Abdoul et al., 2026[1]; Ferdinande and Zanetto, 2026[30]; Zanetto et al., 2022[104]).This is analogous to the platelet function analyzer (PFA)-100, another shear-dependent whole-blood assay, albeit not under controlled flow conditions, in which prolonged closure times are thought to be driven predominantly by thrombocytopenia and anaemia (Balcar et al., 2024[6]). Importantly, impaired thrombus formation under flow may appear contradictory to the observation of increased whole-blood per-platelet aggregation (PLT ratio), however, these results interrogate fundamentally different aspects of primary haemostasis: T-TAS captures the integrated shear-dependent process of thrombus formation on a surface, whereas the PLT ratio demonstrates per-platelet aggregability (Zanetto et al., 2022[104]).

Progressive disease burden and the accompanying increase in systemic inflammation may substantially influence both platelet function and the platelet-VWF axis. As cirrhosis progresses, the haemostatic phenotype becomes increasingly dynamic and is frequently modified by superimposed complications, most notably bacterial infection and acute kidney injury (AKI), both of which contribute to a more unstable clinical state and secondary amplified inflammatory milieu (Zanetto et al., 2020[115], 2022[105], 2023[112]; Zanetto and Simioni, 2023[119]). AKI has been associated with impaired platelet aggregation and secretion, whereas recovery of renal function is accompanied by significant improvements in both parameters, irrespective of the degree of thrombocytopenia (Zanetto et al., 2020[115], 2023[112]). Similarly, previous studies have demonstrated that, in patients with decompensated cirrhosis, bacterial infection is associated with significantly reduced whole-blood platelet aggregation despite comparable platelet counts and no significant differences in VWF levels between infected and matched non-infected patients, suggesting the development of an acquired platelet hypo-aggregative phenotype (Zanetto et al., 2022[105], 2023[112], 2024[109]). Furthermore, resolution of infection has been shown to be accompanied by a further significant decline in platelet aggregation, independent of the severity of thrombocytopenia (Zanetto et al., 2022[105]). A recent study confirmed the presence of platelet hypo-aggregation in patients with decompensated cirrhosis and bacterial infection using thromboelastography (TEG) Platelet Mapping, demonstrating significantly impaired ADP-induced platelet aggregation compared with stable decompensated cirrhosis (Athanasaki et al., 2026[5]). This impairment occurred despite markedly elevated VWF levels, indicating that the increase in VWF does not preserve platelet aggregation in the setting of increasing systemic inflammation (Athanasaki et al., 2026[5]). Moreover, ADP-induced platelet aggregation progressively deteriorated with clinical severity, and was most pronounced in patients with organ failure, while also independently predicting short-term mortality (Athanasaki et al., 2026[5]).

The alterations in platelet function observed with progression of liver disease and superimposed bacterial infection suggest that the haemostatic phenotype of platelets may be substantially modified by systemic inflammation, with consequent effects on platelet function and platelet-VWF interactions (Ferdinande and Zanetto, 2026[30]; La Mura et al., 2026[49]; Zanetto et al., 2023[112]). However, the pathophysiological mechanisms underlying the progressive decline in platelet aggregation under these conditions remain poorly understood. This is particularly intriguing given that, during severe infection and sepsis, platelets are increasingly recognised as non-haemostatic central orchestrators of thromboinflammatory responses through pathogen recognition, crosstalk with innate immune cells, and the induction of neutrophil extracellular trap (NET) formation, thereby contributing to endothelial dysfunction and microvascular thrombosis (Engelmann and Massberg, 2013[28]; Kaiser et al., 2025[48]; Mariotti et al., 2026[59]). Consequently, advanced cirrhosis complicated by severe infection may be characterised by the coexistence of platelet-driven thromboinflammatory activity and impaired haemostatic platelet function (Athanasaki et al., 2026[5]; Ferdinande and Zanetto, 2026[30]; Kaiser et al., 2025[48]; Zanetto et al., 2022[105], 2023[112]). How these co-existing paradoxical platelet functions are linked remains unknown. In particular, it is unclear whether platelets within the thromboinflammatory response ultimately lead to platelet exhaustion, desensitisation, receptor dysfunction, or other qualitative alterations that manifest as impaired aggregation. Future studies should therefore focus on elucidating the mechanisms responsible for reduced platelet aggregation in critically ill patients with cirrhosis and superimposed infection. Equally important will be the correlation of these biological alterations with clinically relevant outcomes, including bleeding complications, thrombosis, organ failure and mortality. A more comprehensive understanding of the interplay between platelet hypofunction and platelet-mediated thromboinflammation may not only improve our understanding of haemostatic dysregulation in advanced infection-associated cirrhosis, but could also uncover novel mechanistic targets for risk stratification and therapeutic intervention in this vulnerable patient population.

### Coagulation

Secondary haemostasis in cirrhosis is characterised by a parallel decline in hepatically synthesised procoagulant factors (II, V, VII, IX, X, XI) and natural anticoagulants (protein C, protein S, antithrombin), while factor VIII, produced by sinusoidal and extra-hepatic endothelial cells, is markedly elevated, driven by endotoxemia-stimulated endothelial cells (Carnevale et al., 2017[14]; Tripodi and Mannucci, 2011[91]). The net effect of these simultaneous changes on thrombin generation has been a central question in the field, and has been most comprehensively addressed through thrombin generation assays. Platelet-poor plasma thrombin generation (PPP-TG) has fundamentally reshaped the understanding of haemostasis in cirrhosis (Lisman et al., 2022[52]; Tripodi et al., 2005[93], 2017[92]; Tripodi and Mannucci, 2011[91]). For more than a decade, a consistent body of evidence has suggested that patients with cirrhosis, including those with advanced disease, retain a preserved capacity to generate thrombin, resulting in the concept of 'rebalanced haemostasis' (Lisman et al., 2022[52]; Lisman and Porte, 2010[54]). These observations diverge from those obtained using TEG and rotational thromboelastometry (ROTEM), which characteristically reveal a progressively hypo-coagulable phenotype with increasing hepatic dysfunction, most notably in ACLF, paralleling systemic inflammation and clinical deterioration (Blasi et al., 2018[8]). However, TEG and ROTEM do not account for VWF or the contribution of protein C; therefore, they may suggest a hypo-coagulable state that does not fully reflect the *in vivo* haemostatic balance, potentially leading to an underestimation of haemostatic potential (Lisman et al., 2022[52]; Northup et al., 2021[68]). Despite thrombomodulin-modified TG performed in PPP being widely regarded as the most comprehensive assay for evaluating the coagulopathy of cirrhosis (Lebreton et al., 2020[51]; Lisman et al., 2022[52]), PPP-based assays exclude circulating blood cells and rely on artificial phospholipid substitutes, conditions that differ substantially from the *in vivo* setting, where coagulation initiation and propagation occur on activated platelet and erythrocyte surfaces (Zanetto et al., 2024[107]). Thrombocytopenia and anaemia may therefore critically influence TG results. For these reasons, whole-blood TG (WB-TG), which incorporates the contributions of circulating plasma elements, may provide a complementary physiologically relevant assessment of coagulation in cirrhosis (Sun et al., 2023[89]; Wan et al., 2020[99], 2021[98]). A recent prospective study compared WB-TG with PPP-TG. In compensated cirrhosis, both assays demonstrated increased endogenous thrombin potential (ETP) relative to controls (Zanetto et al., 2024[107]). In decompensated cirrhosis, however, the assays diverged markedly. PPP-TG continued to indicate a hypercoagulable profile, characterised by elevated ETP, increased peak height, and shortened time-to-peak, reinforcing the traditional paradigm. By contrast, WB-TG revealed progressive impairment of TG kinetics and capacity, culminating in a hypo-coagulable state in Child-Pugh C. This hypo-coagulable phenotype was further exacerbated by bacterial infections, which produced additional reductions in ETP and peak height (Zanetto et al., 2024[107]). Importantly, the divergence between PPP-TG and WB-TG indicates that the plasmatic cellular components exert an important modulatory influence on TG (Zanetto et al., 2024[113], 2025[106]). Platelets and erythrocytes provide phosphatidylserine-exposing membrane surfaces essential for the assembly of tenase and prothrombinase complexes, and their quantitative reduction in advanced cirrhosis likely limits the catalytic surface available for thrombin propagation (Sun et al., 2023[89]; Wan et al., 2021[98]). The more profoundly impaired WB-TG observed in infection-associated cirrhosis compared with non-infected patients warrants further investigation, as it may reflect inflammation-driven alterations in blood cell procoagulant function, consumption of haemostatic factors, or both (Driever and Lisman, 2022[21]; Zanetto et al., 2024[107]).

While the concept of rebalanced haemostasis remains valuable, WB-TG may provide complementary information, especially in decompensated and infection-associated cirrhosis. Whether this translates into clinically actionable risk stratification requires further prospective validation.

### Fibrinolysis

Elevated levels of tissue-type plasminogen activator (tPA) and urokinase-type plasminogen activator (uPA), combined with reduced levels of α2-antiplasmine, thrombin-activatable fibrinolysis inhibitor (TAFI), and factor XIII, promote fibrinolysis, while decreased plasminogen and elevated plasminogen activator inhibitor-1 (PAI-1) provide partial antifibrinolytic compensation (Lisman et al., 2022[52]; Lisman and Porte, 2010[54]). Additionally, preliminary evidence suggests that platelet-derived protease nexin-1 (PN-1), a serine protease inhibitor stored in platelet α-granules, may contribute to fibrinolytic regulation in cirrhosis (Riescher-Tuczkiewicz et al., 2025[81]). Experimental inhibition of PN-1 in whole-blood ROTEM enhanced fibrinolysis in patients with stable decompensated cirrhosis compared to healthy controls, despite no significant differences in plasma or intraplatelet PN-1 concentrations, suggesting that PN-1 counteracts the pro-fibrinolytic effect of elevated tPA and acts synergistically with PAI-1 in maintaining fibrinolytic control (Riescher-Tuczkiewicz et al., 2025[81]). However, these findings are based on small sample sizes limited to stable decompensation and require validation in larger cohorts across the full spectrum of cirrhosis. In stable cirrhosis, the interplay between these pro- and antifibrinolytic alterations results in a rebalanced fibrinolytic state (van den Boom and Lisman, 2022[96]). However, during acute decompensation and ACLF, a mixed fibrinolytic phenotype emerges, a complexity that is further underscored by the heterogeneous results obtained from different functional assays (Blasi et al., 2020[9]).

The plasma-based clot lysis assay, one of the earliest functional methods to assess fibrinolysis, revealed heterogeneity among patients with advanced cirrhosis, with some exhibiting shortened clot lysis times consistent with hyperfibrinolysis, while others displayed normal or hypofibrinolytic profiles (Blasi et al., 2020[9]). In contrast, viscoelastic testing using TEG or ROTEM has generally indicated a normal-to-hypofibrinolytic profile in cirrhosis, although these methods have limited sensitivity for moderate fibrinolytic disturbances (Northup et al., 2021[68]). Clot lysis time is a composite measure influenced by fibrinolytic enzyme activity, clot structure, fibrinogen concentration, and fibrin network permeability, factors that are altered in cirrhosis, especially in the most critically ill cirrhosis patients (Zermatten et al., 2020[121]; Zheng et al., 2023[122]). Fibrin clots from patients with ACLF have been shown to be mechanically weaker and to rupture at lower shear stress than those from healthy controls, and previous conclusions regarding decreased clot permeability in this population may have been confounded by fibrinogen hypersialylation, which leads to underestimation of clot permeability through enhanced water retention (Driever et al., 2023[23]). Shortened clot lysis times in advanced cirrhosis may therefore, at least partly, reflect increased susceptibility of structurally altered fibrin clots to degradation rather than a true increase in fibrinolytic enzymatic activity, although this hypothesis warrants further investigation.

To further characterise the fibrinolytic alterations in cirrhosis, plasmin generation (PG), a novel assay that quantifies the kinetics and amplitude of plasmin formation, was recently applied (Willems et al., 2025[102]). By directly quantifying the kinetics and amplitude of plasmin formation, PG provides a dynamic and integrative assessment of fibrinolytic capacity. Unlike static biomarkers or clot lysis time, PG simultaneously captures both delayed onset and diminished magnitude of fibrinolytic activity. A recent study demonstrated that patients with cirrhosis exhibit a significantly reduced PG capacity, with progressive declines in endogenous plasmin potential and plasmin peak height alongside prolonged lag time with worsening liver disease (Willems et al., 2025[102]). Despite reduced plasmin-generating capacity, clot lysis time was shortened, raising the possibility that the seemingly accelerated clot dissolution observed in advanced cirrhosis may be partly driven by alterations in fibrin clot properties, however this warrants further investigation (Willems et al., 2025[102]). The thrombin-plasmin generation (TG/PG) ratio increased with disease severity, revealing an expanding disequilibrium between coagulation and fibrinolysis in advanced cirrhosis (Willems et al., 2025[102]). These findings illustrate that clot lysis time and plasmin generation assess different aspects of the fibrinolytic process. Given the distinct methodological principles, their results should be interpreted within the context of each individual test. As both assays remain research tools, future studies are required to determine how PG, clot lysis time, and TG/PG profiles relate to clinically relevant outcomes such as bleeding and thrombosis.

Superimposed bacterial infection and sepsis appear to alter the fibrinolytic phenotype in cirrhosis (Blasi et al., 2020[9]). Patients with cirrhosis complicated by sepsis and organ failure have been shown to exhibit prolonged clot lysis times, consistent with a hypofibrinolytic state, while baseline hypofibrinolysis has been associated with increased 30-day mortality (Blasi et al., 2020[9]). These findings suggest that impaired fibrinolytic capacity may contribute to adverse outcomes, potentially through insufficient clearance of intra-organ microthrombi and subsequent progression of organ dysfunction (Blasi et al., 2020[9]). However, the mechanisms underlying this shift towards hypofibrinolysis remain incompletely understood. Emerging evidence from the sepsis literature has recently identified NET-associated neutrophil elastase as a potential mediator of fibrinolytic insufficiency through cleavage of plasminogen into inactive fragments, thereby reducing the availability of functional plasminogen and impairing fibrin-bound PG (Iba et al., 2026[43]; Tschirhart et al., 2026[94]). Whether this NET-plasminogen axis or other inflammation-mediated components contribute to hypofibrinolysis in infection-associated cirrhosis remains to be elucidated. Figure 2(Fig. 2) demonstrates the changes in the haemostatic status across the spectrum of cirrhosis.

## The Link between Haemostasis, Inflammation and Disease Progression in Cirrhosis

Whereas haemostatic and immune pathways were traditionally viewed as independent biological systems, increasing evidence now demonstrates that they operate as an integrated, mutually reinforcing network, leading to the concept of immunohaemostasis and immunothrombosis (Kaiser et al., 2025[48]). Immunohaemostasis describes the physiological interplay between the immune system and haemostasis to maintain vascular integrity and prevent pathogen dissemination, while immunothrombosis refers to the pathological amplification of these mechanisms leading to microvascular thrombosis, driving both protective and detrimental outcomes (Kaiser et al., 2025[48]). In cirrhosis, it has been proposed that intra-hepatic alterations of haemostasis and inflammatory pathways interact and contribute to parenchymal extinction and disease progression (Calvaruso et al., 2008[12]; Hilscher et al., 2019[40]; Lisman and Luyendyk, 2021[53]). The mechanisms responsible for intrahepatic activation of haemostasis are incompletely understood but are likely to involve gut-driven inflammation, promoting sinusoidal cell and platelet activation. However, our understanding of the key molecular mediators that facilitate the crosstalk between inflammation, haemostasis, and disease progression are mostly derived from preclinical models.

### Sinusoidal endothelial dysfunction and immunothrombosis

Liver sinusoidal endothelial cells (LSECs) are highly specialised endothelial cells that, under physiological conditions, maintain an antithrombotic and anti-inflammatory sinusoidal microenvironment through their unique fenestrated architecture, absence of an organised basement membrane, and constitutive production of nitric oxide and anti-adhesive surface molecules (Felli et al., 2026[29]; Gao et al., 2024[33]; Gracia-Sancho et al., 2021[36]). In the context of chronic liver injury, LSECs form the central interface at which haemostatic, immune, and pro-fibrotic pathways seem to converge (Hilscher et al., 2019[40]). LSEC capillarisation, characterised by loss of fenestrae, formation of a basement membrane, and adoption of a continuous endothelial phenotype, precedes and is permissive for hepatic stellate cell (HSC) activation and fibrosis (DeLeve, 2015[18]; Felli et al., 2026[29]). This phenotypic transition is driven by impaired vascular endothelial growth factor (VEGF) -stimulated nitric oxide signalling and is further promoted by hypoxia-induced pathways including Delta-like ligand 4 (DLL4)/Notch1-dependent mechanisms (DeLeve, 2015[18]; Duan et al., 2018[25]; Felli et al., 2026[29]). Capillarised LSECs lose their antithrombotic properties and acquire a pro-adhesive, pro-inflammatory phenotype. Specifically, inflammatory endotheliopathy in LSECs, triggered by harmful hepatic stimuli such as altered gut-liver signalling, pathological shear stress, and other inflammatory mediators, promotes the upregulation of adhesion molecules (ICAM-1, P-selectin, E-selectin), chemokines (chemokine C-X-C motif ligand 1 (CXCL1)), and VWF, collectively facilitating neutrophil recruitment, platelet-neutrophil complex formation, and neutrophil extracellular traps (NET) generation (Felli et al., 2026[29]; Hilscher et al., 2019[40]; Yu et al., 2024[103]). In turn, NETs promote sinusoidal micro-immunothrombosis (Hilscher et al., 2019[40]; Meyers et al., 2022[61]; Yu et al., 2024[103]).

Within this immunothrombotic milieu, HSCs can be activated by thrombin and other coagulation factors through protease-activated receptors PAR-1 and PAR-2 (Gao et al., 2024[33]; Hilscher et al., 2019[40]; Minciuna et al., 2024[64]; Poole et al., 2020[73]; Yu et al., 2024[103]). Transcriptomic profiling has revealed that thrombin induces rapid PAR-1-dependent intracellular calcium mobilisation in HSCs and provokes distinct temporal gene expression programs enriched for cell migration, inflammation, metabolism, and HSC activation signatures, even in the absence of direct upregulation of classic fibrotic mediators. This suggests that thrombin-PAR-1 signalling may prime HSCs for subsequent activation and fibrotic responses through complex transcriptional reprogramming, rather than by immediately inducing fibrotic effector molecules (Mac et al., 2025[56]). Dysfunctional LSECs further amplify this process by failing to maintain HSC quiescence, a function that depends on intact fenestration and paracrine nitric oxide signalling, thereby creating a self-perpetuating cycle of capillarisation, immunothrombosis, and fibrogenesis (Felli et al., 2026[29]). Figure 3(Fig. 3) represents a schematic overview of the molecular and cellular mechanisms linking inflammation, haemostasis and fibrogenesis within the hepatic sinusoidal microenvironment.

### Altered gut-liver axis signalling and immune-vascular crosstalk

In cirrhosis with portal hypertension, disruption of the gut-liver axis is characterised by increased intestinal permeability and enhanced bacterial translocation. This leads to systemic exposure to pathogen-associated molecular patterns, such as lipopolysaccharide (LPS), which drive systemic inflammation and innate immune activation (Zanetto et al., 2023[112]). These processes are exacerbated during episodes of acute decompensation, promoting further decompensation and disease progression (Zanetto et al., 2023[112]). Presepsin, a biomarker reflecting Toll-like receptor activation and systemic inflammation, is significantly elevated in patients with acute decompensation compared to stable decompensated or compensated cirrhosis, and is independently associated with disease progression. These clinical data are consistent with a link between gut-liver axis disruption, innate immune activation, and disease progression (Zanetto et al., 2023[114]). The landmark study by Carnevale and colleagues provided the first evidence that gut-derived LPS directly induces endothelial activation, triggering the release of procoagulant factors VIII and VWF from endothelial cells. Both factors were markedly elevated and strongly correlated with serum LPS. Complementary *in vitro* experiments showed that LPS at clinically relevant concentrations rapidly triggered their release from endothelial cells, an effect abolished by Toll-like receptor 4 inhibition, thereby confirming a causal mechanistic link (Carnevale et al., 2017[14]). Beyond LPS-driven endothelial activation, LPS-associated inflammation arising from a leaky gut also promotes platelet activation within the portal venous district (Queck et al., 2020[76]). This immune-platelet crosstalk in the broader landscape of immunohaemostasis and immunothrombosis, together with inflammation-mediated endothelial activation, is likely to drive the mechanisms described above, ultimately leading to disease progression.

### Non-haemostatic and haemostatic platelet functions in disease progression

Supporting a role for immune-platelet crosstalk in disease progression, platelets in MASLD display a distinct pro-inflammatory transcriptomic profile, consistent with non-haemostatic immunomodulatory functions in disease pathogenesis (Peiseler et al., 2022[72]). Intrahepatic platelet accumulation and enhanced NET formation have been demonstrated in liver biopsies, and both correlate with histological activity and inflammatory severity (Miele et al., 2021[62]). In clinical studies evaluating haemostatic platelet function, increased platelet aggregation, for which earlier studies suggested endotoxin-mediated platelet activation, has been associated with further decompensation and liver-related death in decompensated cirrhosis (Caldwell and Lisman, 2017[11]; Raparelli et al., 2017[77]; Zanetto et al., 2022[104]). Analogous, reticulated platelets (RePLT), 'emergency' platelets released to contrast peripheral destruction, are significantly increased and hyper-activated in cirrhosis patients. Elevated RePLT levels (> 2 %) have been associated with an increased risk of hepatic decompensation and ACLF and may help identify a higher-risk subgroup. (Zanetto et al., 2022[104]). Conversely, when assessing inducible platelet activation, platelet responsiveness to PAR-1 and PAR-4 stimulation, assessed by P-selectin and activated GPIIb/IIIa activation, is reduced in patients with more advanced cirrhosis (Child-Pugh B and C) and severe portal hypertension (HVPG ≥ 20 mmHg) (Brusilovskaya et al., 2023[10]). Preserved inducible platelet activation through these pathways has been reported as an independent prognostic marker of lower risk of hepatic decompensation and mortality (Hofer et al., 2024[41]). These clinical observations demonstrate consistent associations between platelet alterations and outcomes in advanced cirrhosis. Both the observed alterations in non-haemostatic and haemostatic platelet functions support a role for platelets as potential modulators of chronic liver disease progression, pending further investigation to delineate the specific underlying mechanisms.

### Targeting coagulation and platelet pathways to modify disease progression

The aforementioned findings have prompted investigation into whether targeting platelet function or coagulation pathways could influence disease progression. Preclinical data raise the possibility that antiplatelet strategies could have disease-modifying effects in selected liver disease contexts. In a study exploring immune-platelet crosstalk in the context of MASH and hepatocellular carcinoma (HCC), antiplatelet therapy with aspirin, clopidogrel, or ticagrelor reduced intrahepatic platelet accumulation and diminished platelet-immune cell interactions, resulting in attenuation of liver inflammation and injury (Malehmir et al., 2019[57]). These observations suggest that targeting platelet-immune cell crosstalk may represent a promising therapeutic strategy in MASH and its disease progression, although clinical validation is still required (Malehmir et al., 2019[57]). Whether anticoagulation can influence disease progression and the risk of hepatic decompensation, remains an area of investigation. In the IMPORTAL meta-analysis of 500 patients with cirrhosis and portal vein thrombosis (PVT), anticoagulation (low-molecular-weight heparin (LMWH), vitamin K antagonists (VKAs), or direct oral anticoagulants (DOACs)) reduced all-cause mortality independently of recanalisation and was associated with lower liver-related mortality (Guerrero et al., 2023[38]). The CIROXABAN study was a randomised, placebo-controlled trial evaluating rivaroxaban (10 mg daily) in Child-Pugh B7-C10 cirrhosis with portal hypertension (Puente et al., 2025[75]). Although not statistically significant, rivaroxaban lowered the composite endpoint of portal hypertension-related complications, death, or transplantation (26.8 % vs. 46.9 % over 10.1 months), with higher 1- and 2-year event-free survival (78.7 % and 67.1 % vs. 54.4 % and 43 %) (Puente et al., 2025[75]). In a post hoc analysis of Child-Pugh B7 patients, rivaroxaban significantly reduced the risk of decompensation (Puente et al., 2025[75]). Minor bleeding increased, whereas major bleeding did not differ between groups (Puente et al., 2025[75]). More recently, the ApiHep trial evaluated apixaban (5 mg once daily) in a single-centre open-label randomised controlled trial including patients with Child-Pugh B7-C10 cirrhosis without splanchnic venous thrombosis (Agarwal et al., 2026[2]). After a median follow-up of 12 months, apixaban significantly reduced the incidence of new-onset or progressive hepatic decompensation compared with standard of care alone (32.8 % vs. 52.2 %; p = 0.036), while rates of overall bleeding and major bleeding were similar between groups (Agarwal et al., 2026[2]). Trends towards lower rates of ascites and hepatic encephalopathy were also observed, although these did not reach statistical significance (Agarwal et al., 2026[2]). Liver-related mortality did not differ between treatment groups (Agarwal et al., 2026[2]). These findings are compatible with the hypothesis that anticoagulation may influence coagulation-related pathways involved in fibrogenesis and hepatic decompensation. It should be highlighted that, although promising, current evidence does not yet warrant the use of anticoagulants as disease-modifying agents in cirrhosis. Important considerations include prior non-portal hypertensive bleeding and fall risk, particularly in patients with hepatic encephalopathy, frailty or sarcopenia, advanced age, and active alcohol use, all of which increase the risk of traumatic falls. Future research should focus on identifying the patient subgroups most likely to benefit from this therapy, developing individualised benefit-risk algorithms incorporating bleeding history and fall risk, alongside prospective trials to define optimal anticoagulant selection and dosing.

In conclusion, preclinical data support a link between haemostasis, inflammation, and fibrogenesis, while clinical studies provide associative evidence linking haemostatic and inflammatory markers to disease progression in cirrhosis. However, current evidence remains heterogeneous, and the relationship between inflammation-coagulation crosstalk and clinical outcomes is not yet fully understood. Future prospective studies are needed to clarify the temporal dynamics of these pathways, evaluate the predictive value of emerging biomarkers, and identify patients most likely to benefit from targeted interventions. Deeper understanding of the inflammation-coagulation-disease progression crosstalk may reveal novel therapeutic targets and open new avenues for disease modification in cirrhosis.

## Portal Vein Thrombosis and Venous Thromboembolism

PVT represents a frequent and clinically challenging vascular complication in cirrhosis. Meta-analyses report a pooled prevalence of approximately 14 % and a pooled incidence of 5-10 % per 100 patient-years in longitudinal studies (Elkrief et al., 2024[27]). Its occurrence increases proportionally with the severity of hepatic dysfunction, and up to 50 % of cases are diagnosed at the time of liver transplantation (Elkrief et al., 2024[27]; Rautou et al., 2026[78]). Contemporary anticoagulant therapies for PVT achieve complete or partial recanalisation in approximately 60 % of patients; however, nearly one third exhibit no clinically meaningful improvement, and some even experience PVT progression despite treatment (Elkrief et al., 2024[27]). This intriguing observation calls into question the contributory role of systemic hypercoagulability as one of the major primary determinants in the pathophysiology of PVT. Although the pathogenesis of classical venous thrombosis in other vascular beds is inherently multifactorial and traditionally conceptualised through the components of Virchow's triad including stasis, systemic hypercoagulability, and endothelial injury, the portal venous system constitutes a uniquely altered vascular territory, suggesting that local perturbations within the splanchnic circulation play a pivotal role in the PVT development.

### Hypercoagulability

Many studies have tried to assess the specific role of hypercoagulability in the formation of PVT in cirrhosis, while many others have searched for biomarkers that are predictive of *de novo* PVT. The FVIII/PC ratio has been examined as a marker of procoagulant imbalance that increases with advancing liver disease; however, studies comparing this ratio between cirrhotic patients with and without PVT have yielded inconsistent results. Recent rigorous data demonstrate that although the FVIII/PC ratio serves as a predictor of liver-related events and mortality, it is not independently associated with hypercoagulability nor predictive of PVT (Scheiner et al., 2022[83]). Similarly, thrombomodulin resistance, initially described over a decade ago, does not predict PVT but instead reflects the degree of hepatic impairment and parallels the risk of subsequent clinical deterioration (Scheiner et al., 2022[83]). The largest prospective cohort study conducted to date (n = 369; follow-up up to 48 months), incorporating an extensive characterisation of clinical, biochemical, inflammatory, and both acquired and hereditary haemostatic determinants, confirmed substantial alterations across numerous haemostatic parameters, including the FVIII/PC ratio, thrombomodulin resistance, TG, and VWF (Turon et al., 2021[95]). Nonetheless, none of these variables independently predicted *de novo* PVT after accounting for the severity of liver disease (Turon et al., 2021[95]). However, these negative findings should be interpreted in light of important limitations, including the prolonged interval between baseline haemostatic assessment and the occurrence of PVT, the low incidence of PVT limiting statistical power, and the fact that most patients were diagnosed with PVT at the time of decompensation. Consequently, baseline haemostatic measurements may not accurately reflect the dynamic haemostatic changes occurring during the clinical trajectory following study inclusion (Turon et al., 2021[95]). Whereas plasma-based markers of procoagulant imbalance have failed to predict PVT independently of liver disease severity, platelet-function parameters have emerged as independent predictors of PVT development. Prospective data from patients with decompensated cirrhosis demonstrated that increased platelet aggregation, assessed by whole-blood aggregometry, was independently associated with the development of PVT (Zanetto et al., 2023[108]). The platelet ratio demonstrated strong discriminative performance for PVT (AUC 0.92), with a threshold above 0.75 yielding a sensitivity of 83 % and a specificity of 84 %. These findings support its potential utility as a prognostic marker, however, direct insight into the underlying mechanisms of PVT development remains to be proven.

In contrast to cirrhosis without HCC, non-tumoral PVT is considerably more prevalent in cirrhotic patients with HCC, a population in whom a systemic hypercoagulable state is well recognised. HCC drives a shift in haemostasis toward hypercoagulability through several converging mechanisms, including tumour-induced tissue factor expression, enhanced TG, increased platelet aggregation, hypofibrinolysis, and the release of procoagulant microvesicles (Julich-Haertel et al., 2017[47]; Liu et al., 2020[55]; Zanetto et al., 2021[117]). Despite numerous studies evaluating coagulation profiles in cirrhosis with HCC, only a limited number have examined their direct relationship with PVT development. One small study reported that a ROTEM FIBTEM maximal clot firmness (MCF) > 25 mm was associated with a fivefold increase in PVT risk, with both the presence of HCC and elevated ROTEM FIBTEM MCF serving as independent predictors (Zanetto et al., 2017[118]). Although evidence remains limited, these findings suggest that PVT in cirrhosis with HCC may be more influenced by systemic hypercoagulability than in cirrhosis without HCC.

### Haemostasis in the portal circulation

Whether localised hypercoagulability within the portal circulation contributes to PVT formation remains uncertain, as systemic coagulation profiles may not accurately reflect conditions within the portal venous compartment. A comparative analysis of plasma obtained from the portal vein, hepatic vein, and systemic circulation contradicts earlier reports of a distinctly hypercoagulable portal environment, previously described as elevated VWF and factor VIII levels with concomitant reductions in protein C and S (Driever et al., 2022[22]; Praktiknjo et al., 2020[74]). In this study, differences across vascular beds were modest and largely attributable to hepatic clearance (Driever et al., 2022[22]). In contrast, another study investigated the activation of the contact system and intrinsic coagulation pathway in both peripheral and portal venous circulation in decompensated cirrhosis. Markers of the intrinsic pathway activation were significantly higher in portal venous blood than in peripheral blood, suggesting localised activation within the portal circulatory system (Campello et al., 2025[13]). Whether this contributes to PVT formation remains uncertain, particularly because patients with established PVT were not included in these studies.

### Aetiology of liver disease

The aetiology of liver disease may modulate key cellular and molecular processes within the hepatic microenvironment facilitating the development of PVT. A recent national comparative analysis demonstrated that PVT is significantly more prevalent in liver transplant recipients with MASLD compared with those with alcohol-related cirrhosis (14.8 % vs. 9.2 %) (Molinari et al., 2021[65]). Insights from a well-characterised murine MASH model with upregulation of hepatic inflammatory and coagulation pathways demonstrated elevated prothrombin fragment 1+2, thrombin-antithrombin complex, and PAI-1, indicating a prothrombotic state (Pandey et al., 2024[70]). Nevertheless, evidence for systemic hypercoagulability in human MASH cohorts remains limited, except for elevated plasma levels of PAI-1 in patients with MASH cirrhosis, potentially reflecting a prothrombotic state and impaired fibrinolysis (Zanetto et al., 2021[116], 2022[111]). This divergence suggests that, rather than systemic hypercoagulability, the inflammatory hepatic microenvironment in MASH may contribute to PVT susceptibility.

### Systemic inflammation, infection and altered gut-liver axis

Systemic inflammation constitutes another factor implicated in the modulation of coagulation. Experimental evidence demonstrates that NETs generated through Gasdermin D-dependent neutrophil activation, promote portal vein immunothrombosis, thereby linking systemic inflammation to PVT formation (Che et al., 2024[15]). Clinical studies are consistent with this concept but remain associative: in a retrospective cohort, markers of systemic inflammation, including an elevated neutrophil-to-lymphocyte ratio and increased C-reactive protein, were independently associated with the presence of PVT (Huang et al., 2020[42]). Prospective data further indicate that higher interleukin-6 concentrations and lymphopenia independently predict PVT development, independent of portal hypertension severity (Nery et al., 2021[66]). However, findings from a recent large prospective cohort assessing NET levels did not demonstrate an association between cirrhosis-related systemic inflammation and PVT formation (Turon et al., 2021[95]). Interestingly, recent studies have investigated the association between bacterial infection and increased risk of PVT. A prospective cohort study demonstrated that a history of bacterial infection was independently associated with subsequent development of PVT (Dalbeni et al., 2024[16]). A large post-hoc analysis further examined this relationship and found that, while bacterial infection overall did not significantly increase the 6-month risk of PVT in the entire cirrhotic cohort, urinary tract infections were independently associated with higher PVT risk (Di Cola et al., 2025[19]). This association was also reported in an independent prospective cohort, in which prior bacterial infection was significantly associated with *de novo* PVT development (Zanetto et al., 2024[110]).

### Portal vein endotheliopathy

A reduced portal vein flow velocity below 15 cm/s is a contributor to the pathogenesis of PVT, however not confirmed in all studies (Giri et al., 2023[35]; Nery et al., 2015[67]; Turon et al., 2021[95]). Reduced blood flow, leading to diminished shear stress, is a recognised trigger of endothelial dysfunction in various vascular beds; however, the specific consequences of decreased portal venous flow on portal vein endothelial integrity remain incompletely defined and largely speculative (Wei et al., 2017[101]). In contrast to the extensively studied hepatic sinusoidal endothelium, portal vein endothelial cells (PVECs) constitute a largely unexplored vascular territory. The study of PVECs is inherently challenging, as the portal venous system is inaccessible to non-invasive sampling, and histological material can only be obtained at the time of liver transplantation. Nevertheless, significant advances have recently been achieved in characterising this elusive endothelial niche. A recent study has provided detailed mechanistic insights into the pathogenesis of PVT through integrated histological and transcriptomic analyses (Anton et al., 2025[4]). Building on earlier evidence demonstrating that PVT in cirrhosis is predominantly composed of intimal fibrosis, with a superimposed fibrin-rich thrombus present in only approximately one-third of cases, this study offers significant advancements in understanding the pathophysiology of PVT in cirrhosis (Driever et al., 2022[24]). Histological examination of portal vein tissue obtained from cirrhotic explants with and without PVT, as well as from healthy donor livers, revealed that PVT is marked by pronounced eccentric intimal thickening (Anton et al., 2025[4]). Complementary transcriptomic profiling of PVECs isolated from patients with PVT demonstrated significant upregulation of the endothelial-to-mesenchymal transition (EndMT) pathway (Anton et al., 2025[4]). EndMT, a process whereby endothelial cells progressively lose canonical endothelial markers and acquire mesenchymal and myofibroblast-like characteristics, was identified as a key driver of intimal hyperplasia and fibrosis within the portal vein (Anton et al., 2025[4]). Both intimal thickening and transcriptional signatures of EndMT were observed not only at the site of thrombosis but also in portal vein segments distant from the thrombus, indicating a diffuse endothelial remodelling process rather than a purely localised lesion (Anton et al., 2025[4]).

### Aetiological drivers of endothelial-to-mesenchymal transition

Portal hypertension, a known risk factor for PVT, has been proposed as a trigger for EndMT by reducing portal vein blood velocity and exposing PVECs to injurious low shear stress. Although direct evidence is limited, this concept is supported by a study showing that clinical markers of portal hypertension, such as prior variceal bleeding, are associated with portal vein intimal thickening, consistent with maladaptive vascular remodelling induced by elevated portal pressure (Driever et al., 2025[20]). Altered gut-liver axis signalling represents another important potential trigger, promoting a pro-inflammatory portal environment. Elevated levels of inflammatory mediators (LPS, tumour necrosis factor-α, interleukin-6, NETs) have been reported in portal compared with hepatic venous blood, although this gradient was partly attributed to hepatic clearance (Driever et al., 2022[22]). Nevertheless, other evidence supports a distinct pro-inflammatory portal territory. Portal venous endotoxemia exceeds systemic levels, implicating gut-derived inflammatory stimuli, and advanced cirrhosis is associated with marked portal endothelial damage, reflected by increased glycosaminoglycans and endothelial-derived microparticles in portal versus peripheral blood (Shalaby et al., 2020[85]). Further support comes from a portal-specific miRNA signature, characterised by increased miR-21-5p and reduced miR-146a-5p and miR-155-5p, indicative of chronic, dysregulated local inflammation and impaired counter-regulation, accompanied by portal NLRP3 upregulation consistent with inflammasome activation in portal vein endothelium (Ferdinande et al., 2026[31]). In line with this, inflammatory infiltrates (CD68^+^ macrophages and CD3^+^ T cells) have been demonstrated in the portal vein wall of cirrhotic patients, with higher cell numbers in those with PVT, and transcriptomic analyses of cirrhotic PVECs revealed predominant dysregulation of immune-activation and pro-inflammatory pathways (Anton et al., 2025[4]). A third etiological hypothesis implicates localised activation of coagulation within the portal venous district (Campello et al., 2025[13]; Driever et al., 2022[22]; Praktiknjo et al., 2020[74]).

These findings redefine PVT in cirrhosis as a process primarily driven by maladaptive endothelial remodelling, potentially triggered by portal haemodynamic alterations, gut-derived inflammatory stimuli, and localised coagulation activation, in which intimal hyperplasia and fibrosis create a microenvironment permissive to secondary thrombus formation. Beyond anticoagulation for PVT, therapeutic strategies should aim to restore portal vein patency by targeting underlying vascular maladaptation. *In silico* drug repurposing identified statins as potential EndMT-targeting agents, a finding supported by *in vitro* data showing attenuation of EndMT features in primary human PVECs, suggesting a role for statins in preventing or reversing maladaptive intimal remodelling and portal vein obstruction in cirrhosis (Anton et al., 2025[4]). Future research should focus on translational studies and rigorous clinical trials to determine whether pharmacological modulation of endothelial remodelling, including statins and gut-liver axis modulation, can prevent, stabilise, or reverse portal venous obstruction, and to define optimal timing and patient selection. Figure 4(Fig. 4) represents the pathophysiology of PVT in cirrhosis.

### Venous thromboembolism

In patients with cirrhosis without HCC, the risk of venous thromboembolism (VTE) is markedly increased, with an adjusted hazard ratio of 2.0 and annual incidence rates of 0.5-1.9 % in population-based studies (Jepsen et al., 2021[46]; Subhani et al., 2022[88]). In hospitalised patients, the absence of pharmacological prophylaxis further increases VTE risk nearly threefold (Subhani et al., 2022[88]). When VTE does occur, outcomes are significantly worse: data from the RIETE registry show an all-cause mortality of 10.7 % in cirrhotic patients with VTE compared with 3.4 % in those without (Bikdeli et al., 2019[7]). Independent risk factors for in-hospital VTE include inflammation-related variables (elevated CRP, bacterial infections), markers of disease severity (higher Child-Pugh class, AKI), and patient-level factors (male sex, reduced mobility, family history of thrombosis) (Ambrosino et al., 2017[3]; Zanetto et al., 2025[120]). The Cirrhosis Thrombosis Model (CiThroModel), which integrates these determinants, outperforms the Padua score for VTE risk stratification in cirrhosis, with an AUROC of 0.88 versus 0.74 (Zanetto et al., 2025[120]). However, the CiThroModel was developed and internally validated in a single-centre retrospective cohort. External validation in independent, multicentre populations is needed to confirm its discriminative performance and to determine whether it can reliably guide thromboprophylaxis decisions. Recent prospective data in patients with acute decompensation further underscore the central contribution of inflammation in driving VTE risk. Although TG is increased in this setting, neither TG nor other laboratory markers of hypercoagulability predict VTE, whereas elevated CRP levels and high PAI-1 concentrations (> 50 ng/mL) are associated with one-year VTE risk (Zanetto et al., 2023[113]). These findings indicate that inflammatory burden, rather than baseline coagulation parameters, is a major driver of VTE risk and should therefore inform risk assessment. Meta-analyses, largely based on retrospective data, suggest that pharmacological prophylaxis does not significantly increase bleeding risk in cirrhosis; however, its net clinical benefit remains to be established (Pasta et al., 2023[71]). Pending definitive evidence, an individualised approach guided by validated risk stratification tools is warranted (Zanetto et al., 2025[120]).

## Procedure-Related Bleeding in Cirrhosis

### Procedure-related factors

Patients with cirrhosis experience procedure-related bleeding in 6.9 % of admissions and 3.0 % of procedures (Intagliata et al., 2023[45]). Current AASLD and EASL guidelines stratify procedural bleeding risk based on population-level incidence to guide periprocedural haemostatic management, but do not incorporate patient-specific determinants (Northup et al., 2021[68]; Villa et al., 2022[97]). Despite applying the same major bleeding threshold (≥1.5 %), the two guidelines classify several procedures differently, for example, TIPS is considered low-risk by EASL but high-risk by AASLD (Northup et al., 2021[68]; Villa et al., 2022[97]). A recent multispecialty expert consensus involving 52 international experts harmonised bleeding-risk classification for 80 invasive procedures in cirrhosis, reaching consensus for 52: 17 high-risk (mainly interventional endoscopic procedures, percutaneous organ biopsies, and central nervous system procedures) and 35 low-risk (mainly diagnostic procedures), while the lack of consensus for the remaining 28 reflects ongoing uncertainty (Riescher-Tuczkiewicz et al., 2024[80]). Beyond procedure-specific classification, risk stratification should incorporate the feasibility of achieving effective local haemostasis, including selective embolization or local compression, as well as procedural technique and operator expertise, both of which are important but difficult-to-quantify determinants of bleeding risk (Davis et al., 2025[17]; Northup et al., 2021[68]). Imaging guidance, particularly real-time ultrasound, reduces vascular injury during paracentesis, thoracentesis, and TIPS (Mercaldi and Lanes, 2013[60]; Northup et al., 2021[68]), while operator familiarity with the anatomical and haemostatic challenges of advanced cirrhosis, including the ability to adapt or abort procedures and manage post-procedural bleeding, represents a modifiable determinant of bleeding risk (Miller et al., 2025[63]; Northup et al., 2021[68]).

### Patient-related factors

A comprehensive risk assessment should, however, extend beyond procedure-related factors to incorporate the patient's clinical trajectory. The PROC-BLeeD study reported a higher prevalence of ACLF among patients experiencing procedural bleeding compared with those without (42.1 % vs. 19.7 %), supporting the relevance of disease severity at the time of the procedure (Intagliata et al., 2023[45]). Furthermore, patients with acute decompensation and ACLF frequently undergo multiple procedures during a single hospitalisation, and the cumulative procedural burden may confer a higher aggregate bleeding risk than any individual procedure alone. Equally important is the individual bleeding phenotype: a history of prior non-portal hypertensive bleeding serves as a practical indicator of patient-specific bleeding vulnerability. Finally, advanced portal hypertension leads to profoundly altered abdominal vascular anatomy, an often underappreciated factor that may complicate even traditionally low-risk procedures such as paracentesis, particularly in patients with extensive abdominal wall collateralisation requiring repeated interventions.

### Haemostatic assessment

A pivotal shift in preprocedural planning has been the recognition that conventional parameters such as INR prolongation and thrombocytopenia are poor predictors of procedure-related bleeding; contemporary guidelines accordingly discourage routine correction of baseline coagulation abnormalities and uniformly advise against INR correction prior to high-risk procedures (Northup et al., 2021[68]; Riescher-Tuczkiewicz et al., 2024[80]; Villa et al., 2022[97]). Expert consensus suggests minimum acceptable platelet counts of 30 × 10^9^/L for low-risk and 50 × 10^9^/L for high-risk procedures (Riescher-Tuczkiewicz et al., 2024[80]), with EASL recommending individualised consideration of platelet transfusion or thrombopoietin receptor agonists below 50 × 10^9^/L for high-risk interventions (Villa et al., 2022[97]). However, available evidence does not demonstrate a causal relationship between severe thrombocytopenia (< 50 × 10^9^/L) and procedural bleeding, nor a benefit of prophylactic correction, although interpretation is constrained by limited statistical power (Davis et al., 2025[17]; Zanetto et al., 2024[109]). Global haemostatic assays such as TEG and ROTEM offer a more integrative assessment, and their principal clinical value lies in consistently reducing periprocedural transfusion requirements without increasing bleeding complications, thereby reassuring proceduralists of preserved haemostatic competence despite markedly prolonged INR values (Hartmann et al., 2023[39]). Whole-blood thrombin generation may further refine risk stratification: in acute decompensation, an ETP below 350 nmol/L·min accurately predicted major bleeding (AUC 0.854), while values above this threshold demonstrated a negative predictive value of 100 % for major bleeding, including patients with platelet counts below 50 × 10^9^/L (Zanetto et al., 2025[120]). These findings suggest that functional assays may identify patients in whom prophylactic haemostatic interventions can be safely withheld, but methodological standardisation and external validation remain essential before clinical implementation. Bacterial infection, AKI, and severe anaemia each independently alter platelet function, secondary haemostasis, and fibrinolytic pathways, contributing to an unstable haemostatic milieu that may persist beyond apparent clinical resolution (Athanasaki et al., 2026[5]; Lassila and Weisel, 2023[50]; Zanetto et al., 2020[115], 2022[105], 2023[112]). WB-TG is significantly reduced in infected compared with non-infected patients (Zanetto et al., 2024[107], 2025[106]). Similarly, despite comparable platelet counts, whole-blood platelet aggregation is lower in infected than in non-infected patients, highlighting that functional platelet impairment driven by systemic inflammation is not captured by platelet count alone (Athanasaki et al., 2026; Zanetto et al., 2024[107], 2025[106]). These observations underscore the limitations of rigid, numerically defined thresholds and support a multidimensional approach to bleeding risk assessment that integrates clinical trajectory, procedural characteristics, systemic inflammatory modifiers, and functional haemostatic assays, while accounting for the dynamic nature of haemostasis where baseline testing may rapidly become obsolete with clinical deterioration. Figure 5(Fig. 5) provides an overview of the multidimensional factors informing periprocedural risk assessment in cirrhosis.

## Conclusion

Haemostasis in cirrhosis is a dynamic entity characterised by simultaneous alterations in platelets, coagulation, and fibrinolysis that become more pronounced with disease progression, systemic inflammation, and superimposed complications. The demonstration through PPP-TG that patients with cirrhosis retain thrombin-generating capacity established the paradigm of rebalanced haemostasis. More recently, advanced functional assays have since provided complementary perspectives: whole-blood platelet aggregometry reveals increased per-platelet aggregation in decompensated cirrhosis, while bacterial infection induces an acquired hypo-aggregative phenotype that may paradoxically coexist with platelet-driven thromboinflammatory activity. Whole-blood TG demonstrates progressive hypocoagulability in decompensated disease, underscoring the modulatory influence of thrombocytopenia and anaemia. The plasmin generation assay suggests a reduction in fibrinolytic capacity with advancing disease, whereas shortened clot lysis times have also been reported. Since these tests are based on different principles and assess different aspects of the fibrinolytic system, their results should not be considered interchangeable. However, no single test currently captures the full haemostatic complexity, and the rapidly changing clinical trajectory of advanced liver disease renders bleeding risk assessment particularly challenging. A multidimensional risk assessment is therefore proposed, integrating procedure-related factors, clinical trajectory, systemic inflammation, functional haemostatic testing, and intercurrent modifiers such as infection, anaemia, and AKI.

Preclinical evidence increasingly supports an integrated paradigm in which haemostasis, inflammation, and liver disease progression are intricately interconnected. Altered gut-liver axis signalling, considered one of the key upstream triggers, may drive LSEC capillarisation, endothelial and platelet activation, sinusoidal immunothrombosis through neutrophil recruitment and NET formation, and thrombin-driven hepatic stellate cell activation, collectively promoting fibrogenesis and disease progression. Clinical data provide associative support; platelet- and coagulation-derived biomarkers are independently associated with hepatic decompensation and mortality, while early signals from anticoagulation trials suggest that targeting coagulation pathways may reduce the risk of decompensation. Deeper elucidation of the inflammation-coagulation-fibrogenesis axis may therefore reveal novel therapeutic targets and open new avenues for disease modulation, although current evidence does not yet warrant the use of anticoagulants or antiplatelet agents as disease-modifying therapy outside clinical trials.

The pathophysiology of PVT is increasingly being reconceptualised as predominantly a vascular remodelling disorder driven by EndMT. Portal hypertension-induced low shear stress, inflammation, altered gut-liver axis signalling, localised coagulation activation, and MASLD-associated inflammatory pathways have emerged as potential drivers of EndMT. The resulting intimal hyperplasia and fibrosis may establish a microenvironment permissive to secondary thrombus formation. Further elucidation of the mechanisms underlying EndMT may identify novel therapeutic targets aimed at preventing, stabilising, or reversing portal vein remodelling.

Further studies investigating haemostasis in cirrhosis and the haemostasis-inflammation interface will improve our understanding of cirrhotic coagulopathy and its clinical implications, and may ultimately inform new strategies for risk stratification and therapeutic targets in advanced chronic liver disease.

## Notes

Alberto Zanetto and Kymentie Ferdinande contributed equally as first author.

Alberto Zanetto and Marco Senzolo (Department of Surgery, Oncology and Gastroenterology, University of Padova, Via Nicolò Giustiniani 2, 35128 Padova, Italy; E-mail: marcosenzolo@hotmail.com) contributed equally as corresponding author.

## Declaration

### Author contributions

All authors contributed to review idea, writing and revision. MS: final approval.

### Conflict of interest

All authors declare to have no conflicts of interest.

### Artificial Intelligence (AI) - assisted technology

No artificial intelligence was used in creating the content or figures of this manuscript.

### Data availability statement

No new data were generated in this review.

### Financial support

None

### Trial registration number

NA

## Figures and Tables

**Figure 1:**
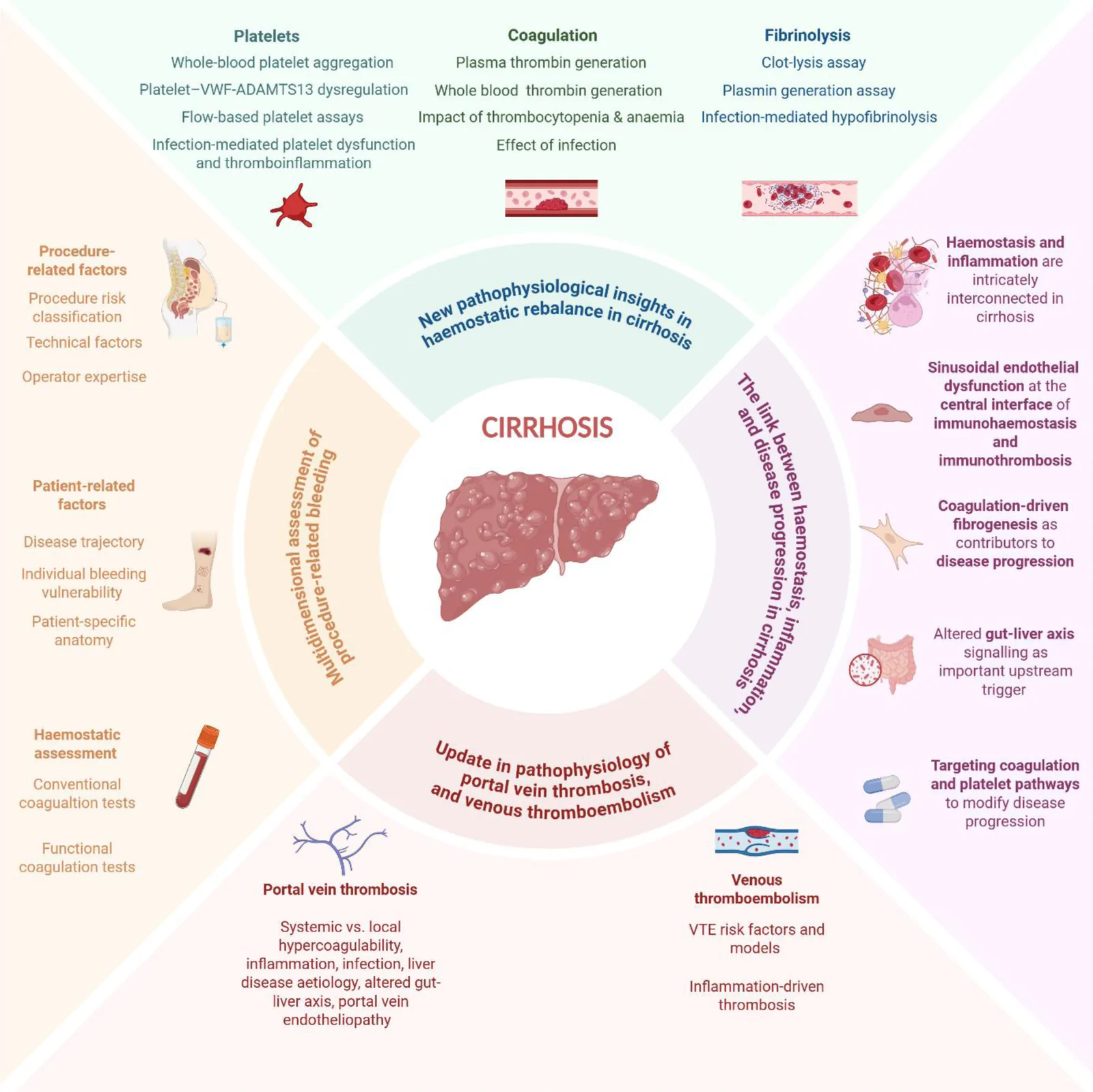
Graphical abstract

**Figure 2:**
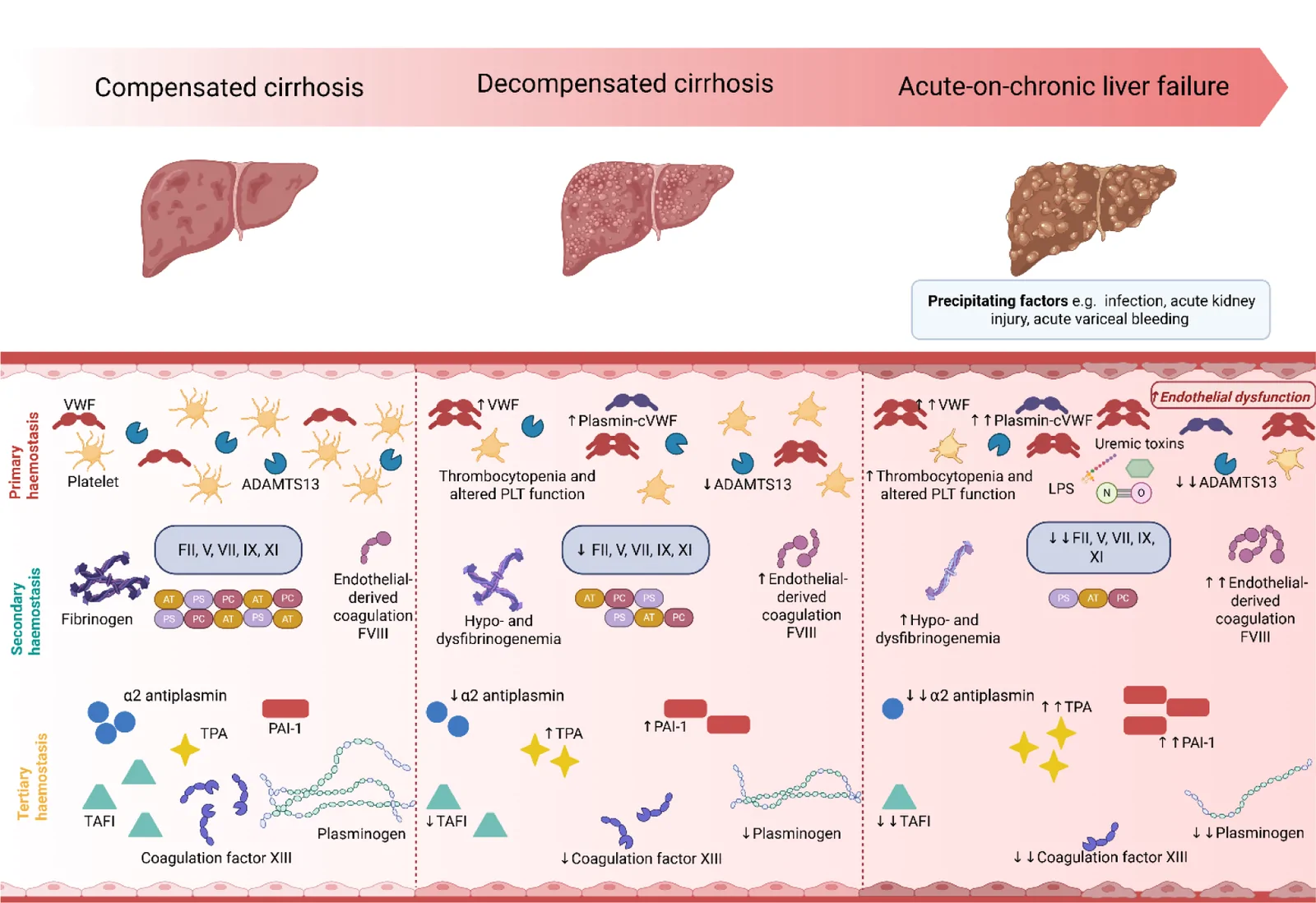
Changes in the haemostatic balance across the spectrum of cirrhosis. This schematic illustrates the evolving haemostatic status from compensated cirrhosis through decompensated cirrhosis to acute-on-chronic liver failure (ACLF). Progression of liver disease is associated with profound, multifactorial disturbances in primary, secondary, and tertiary haemostasis, resulting in a fragile and highly dynamic haemostatic equilibrium. In compensated cirrhosis, haemostasis is largely preserved through a balanced reduction of procoagulant (Factor (F) II, V, VII, IX, X, XI) and anticoagulant (protein C (PC), protein S (PS), and antithrombin (AT)) factors, with relatively maintained platelet-von Willebrand factor (VWF) interactions and fibrinolytic activity. With progression to decompensated cirrhosis, increasing endothelial dysfunction and systemic inflammation drive rising levels of endothelial-derived VWF including high-molecular-weight VWF, together with elevated factor VIII and reduced activity of the VWF-cleaving protease ADAMTS13. Alternative VWF cleaving mechanisms include plasmin-cleavage leading to plasmin-cleaved VWF fragments (plasmin-cVWF). These changes are accompanied by thrombocytopenia, altered platelet function, declining levels of procoagulant coagulation factors and natural anticoagulants, as well as quantitative and qualitative fibrinogen abnormalities. In parallel, progressive disturbances in fibrinolysis emerge, characterised by reduced plasminogen levels, increased tissue plasminogen activator (tPA), reduced levels of the plasmin inhibitor α2-antiplasmin, decreased thrombin-activatable fibrinolysis inhibitor (TAFI), increased plasminogen activator inhibitor-1 (PAI-1), and reduced levels of the fibrin-stabilising factor XIII. In ACLF, these haemostatic derangements become more severe and are further amplified, resulting in a profoundly unstable haemostatic state. The haemostatic balance is dynamically modified by destabilising factors, including bacterial infection and acute kidney injury (AKI). These haemostatic-modifying factors act as modulators capable of shifting the haemostatic equilibrium toward either hypo- or hypercoagulability. In the setting of bacterial infection, nitric oxide (NO) levels are markedly increased and exert inhibitory effects on platelet adhesion and aggregation through activation of soluble guanylate cyclase and increased intracellular cyclic guanosine monophosphate (cGMP). Lipopolysaccharide (LPS), a component of the outer membrane of Gram-negative bacteria, translocates into the systemic circulation due to increased gut permeability, triggering systemic inflammation, endothelial activation, upregulation of tissue factor, and alterations in platelet aggregation. In AKI, accumulation of uremic toxins due to impaired renal clearance induces endothelial dysfunction through oxidative stress and inflammatory pathways, promotes expression of prothrombotic mediators such as tissue factor, and further impairs platelet function.

**Figure 3:**
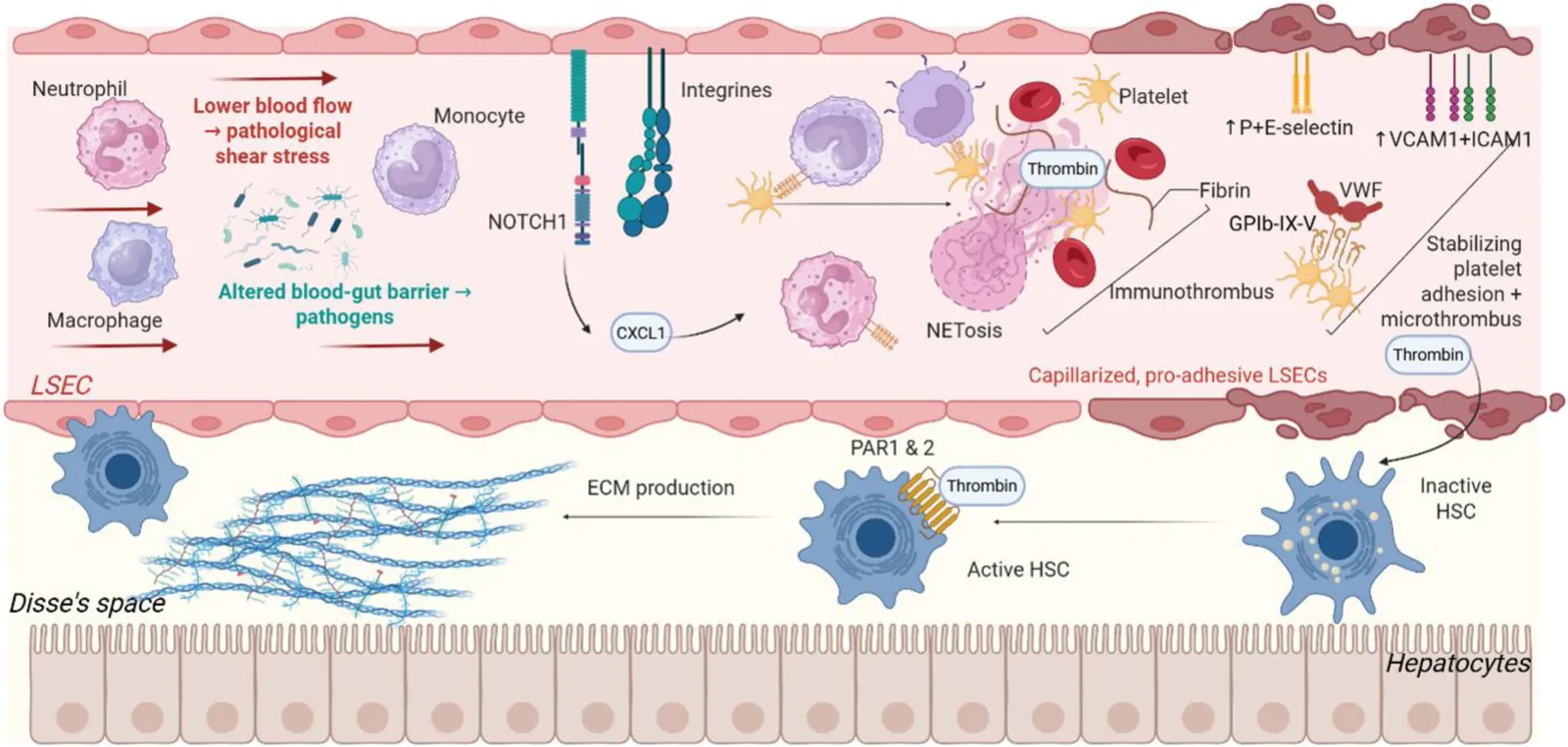
Crosstalk between inflammation, coagulation, and disease progression in chronic liver disease. Schematic overview of the molecular and cellular mechanisms linking inflammation, haemostasis, and fibrogenesis within the hepatic sinusoidal microenvironment. Harmful hepatic stimuli, including altered gut-liver signalling with pathogen translocation, reduced sinusoidal blood flow resulting in pathological shear stress, and inflammatory mediators, reprogramme liver sinusoidal endothelial cells (LSECs) from an antithrombotic state towards a capillarised, pro-adhesive phenotype. This phenotypic shift is mediated through integrin- and Notch1-dependent signalling pathways and is associated with increased expression of the chemokine C-X-C motif ligand 1 (CXCL1), promoting neutrophil recruitment to the sinusoid. This process facilitates platelet-neutrophil interactions, neutrophil extracellular trap (NET) formation, fibrin deposition, and immunothrombus development within the sinusoidal lumen. Concurrent increases in P-selectin, E-selectin, vascular cell adhesion molecule 1 (VCAM-1), intercellular adhesion molecule 1 (ICAM-1), and von Willebrand factor (VWF)- platelet engagement via the glycoprotein Ib-IX-V complex (GPIb-IX-V), stabilise platelet adhesion and promote sinusoidal micro-immunothrombosis. Within this immunothrombotic microenvironment, thrombin generation and other coagulation proteases may activate hepatic stellate cells (HSCs) via protease-activated receptors PAR-1 and PAR-2, ultimately leading to increased extracellular matrix (ECM) production in the space of Disse.

**Figure 4:**
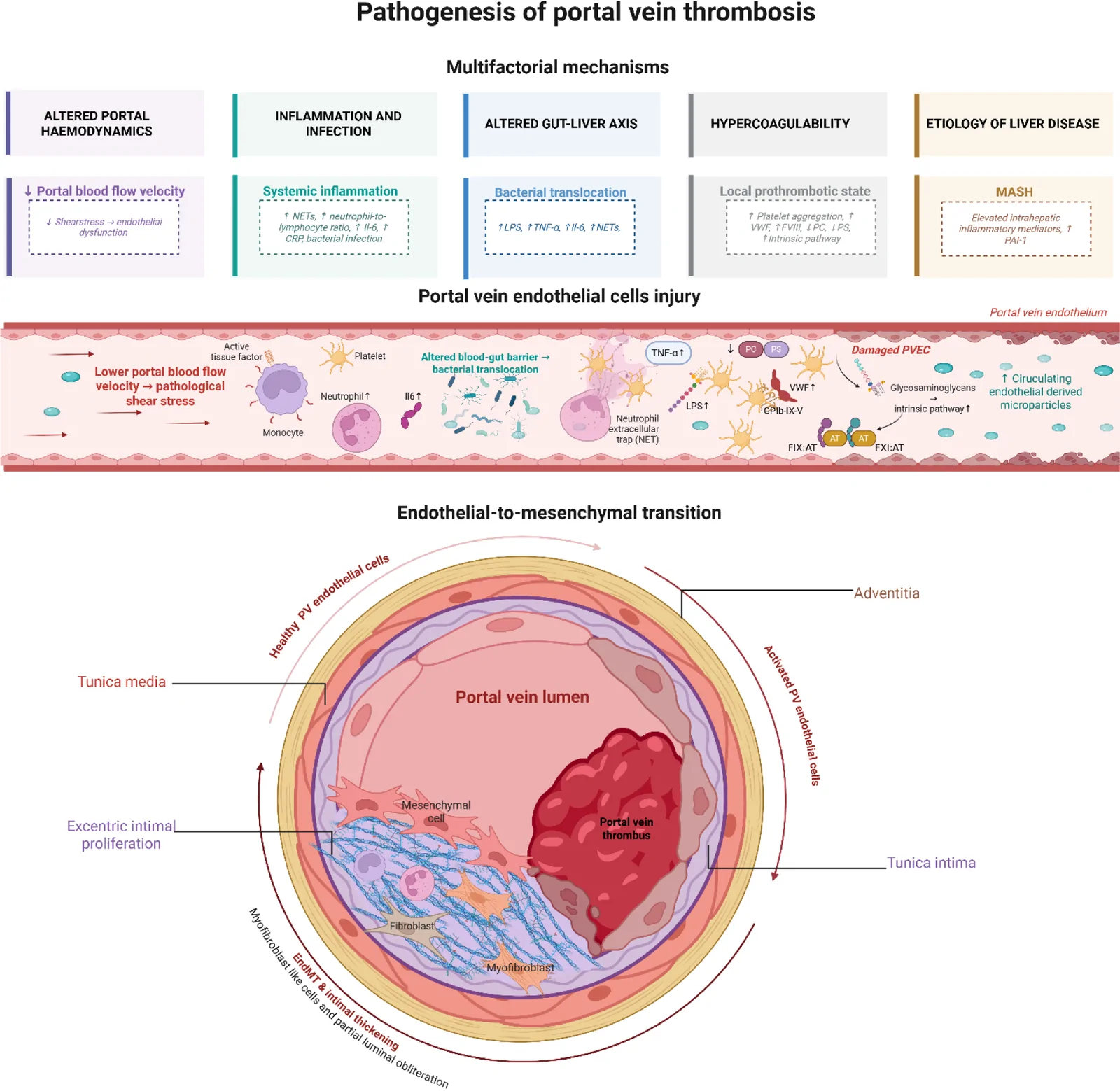
Pathogenesis of portal vein thrombosis. This figure depicts the multifactorial mechanisms contributing to the development of portal vein thrombosis (PVT) in cirrhosis. Altered portal haemodynamics, characterised by reduced blood flow velocity and pathological shear stress, promote endothelial dysfunction. Disruption of the gut-liver axis facilitates bacterial translocation, characterised by increased inflammatory mediators, including lipopolysaccharide (LPS), interleukin 6 (IL-6), and tumour necrosis factor-α (TNF-α), which drive endothelial activation, leukocyte recruitment, and neutrophil extracellular trap (NET) formation. A local prothrombotic milieu is characterised by increased platelet aggregation, release of endothelial-derived von Willebrand factor (VWF), generation of endothelial-derived microparticles, and deficiencies in the anticoagulant protein C-protein S pathway. Alterations of the endothelial glycocalyx, rich in glycosaminoglycans, promote activation of the intrinsic coagulation pathway, with local generation of factor XI- and factor IX-antithrombin-bound complexes (FXIa:AT and FIXa:AT), suggesting a portal-specific, heparin-like effect mediated by the damaged endothelial surface. The aetiology of the underlying liver disease, such as metabolic dysfunction-associated steatohepatitis (MASH), may further exacerbate intrahepatic inflammation and prothrombotic signalling. These converging mechanisms culminate in portal vein endothelial cell injury and activation. Central to PVT pathogenesis is endothelial-to-mesenchymal transition (EndMT), leading to intimal proliferation, vascular remodelling, eccentric luminal narrowing, and ultimately portal vein thrombus formation.

**Figure 5:**
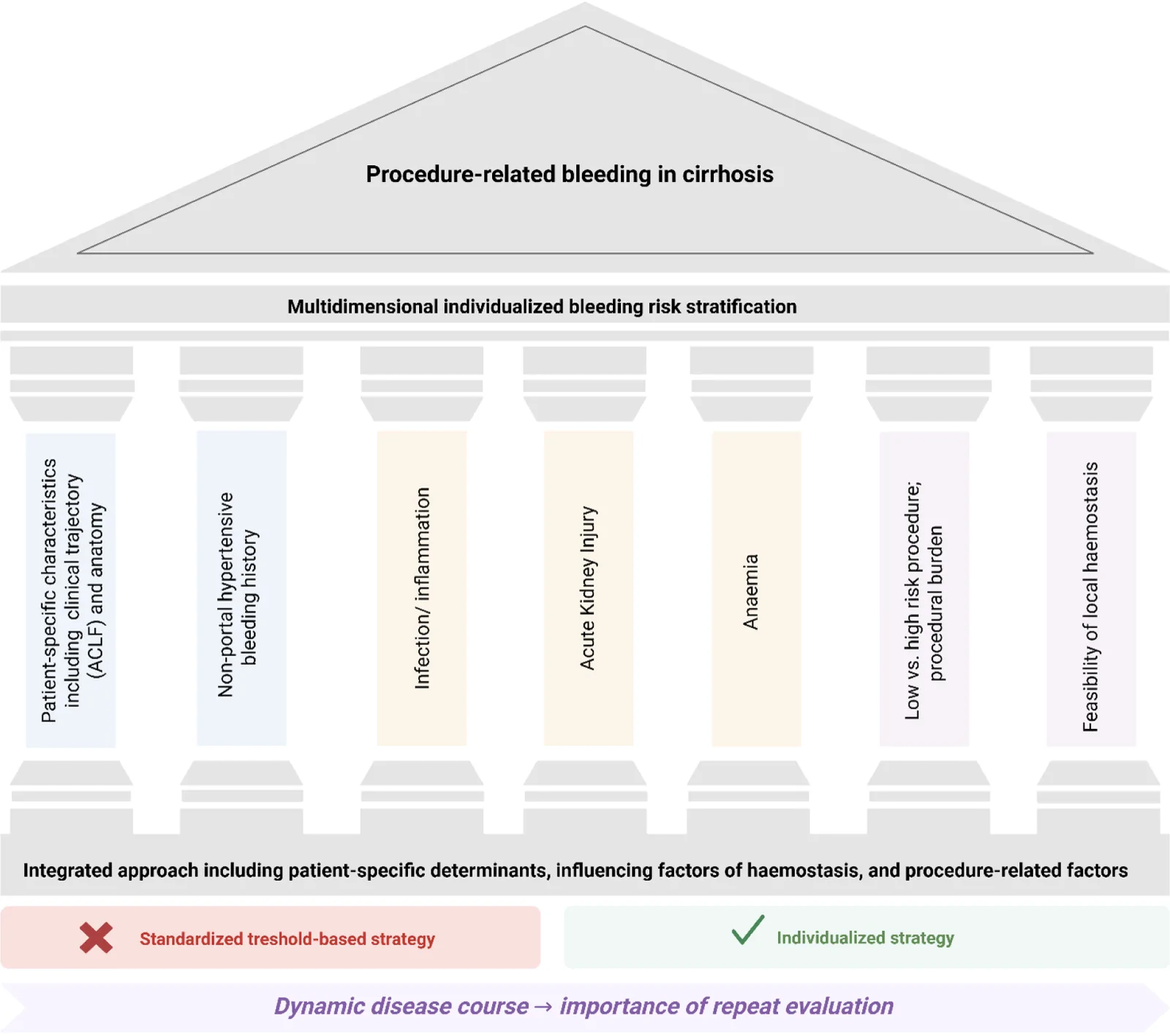
Periprocedural bleeding risk assessment in cirrhosis. This schematic illustrates a multidimensional and individualised approach to periprocedural bleeding risk assessment in patients with cirrhosis. Procedure-related bleeding risk arises from the dynamic interplay between patient-specific characteristics (blue), influencing factors of haemostasis (yellow), and procedure-related factors (purple), which together support an integrated risk stratification framework. Patient-specific characteristics include the individual clinical trajectory, such as acute-on-chronic liver failure (ACLF), patient-specific vascular anatomy, and a history of non-portal hypertensive bleeding. Influencing factors of haemostasis comprise infection or systemic inflammation, acute kidney injury, and anaemia. Procedure-related factors include the intrinsic bleeding risk of the procedure (low vs. high risk), the total procedural burden, and the feasibility of achieving effective local haemostasis. These complementary dimensions collectively underpin a personalised bleeding risk assessment strategy, emphasising the limitations of standardised, threshold-based approaches and highlighting the need for an individualised, clinically integrated evaluation. Given the dynamic course of cirrhosis, repeated reassessment over time and in response to changes in clinical status is essential.
